# Supplementary material for: Complexome profiling of the Chlamydomonas psb28 mutant reveals TEF5 as an early PSII assembly factor
Source: Plant Cell. 2025 Mar 17;37(6):koaf055. doi: 10.1093/plcell/koaf055 (PMC12164590; doi:10.1093/plcell/koaf055)
Supplement: koaf055_Supplementary_Data [file koaf055_supplementary_data.zip › psb28_interactive.zip!/plots/intensity/qualityPlots/peptides/_Cre17.g702900.t1.2.html]

### Cre17.g702900.t1.2

Trivial name:   
SeqCoverage [median]: 19.80  
Mass: 24.28 kDa  
  
 

| Sequence | MissedCleavage | Mass [Da] | wtIntensity | muIntensity | Score |
| --- | --- | --- | --- | --- | --- |
| AQLAQMAPVVK | 0 | 1154.65 | 0.00 | 2621.38 | 129.42 |
| AQLAQMAPVVKDEWVR | 1 | 1839.97 | 493.43 | 5097.35 | 85.62 |
| AQTHHTMDVESLSSSEGK | 0 | 1942.87 | 1485.04 | 8302.64 | 103.41 |
|  |  |  | 1978.5 | 16021.4 |  |
